# A Suggested Treatment for Functional Aphonia
1Read at a Meeting of the Bristol Medico-Chirurgical Society at Weston-super-Mare, on June 12th, 1907.


**Published:** 1907-09

**Authors:** C. Percival Crouch

**Affiliations:** Hon. Surgeon, Weston-Super-Mare Hospital.


### A SUGGESTED TREATMENT FOR FUNCTIONAL APHONIA.[1]

BY

C. PERCIVAL CROUCH, M.B. (Lond.), F.R.C.S.
*Hon. Surgeon, Weston-super-Mare Hospital.*

---

THE treatment of functional aphonia has always struck me as being unsatisfactory.

In the majority of cases, of course, the voice can, so to speak, be frightened back. The introduction of a mysterious instrument, as the laryngoscopic mirror must appear to the uneducated mind, in many cases serves the purpose. In other cases the more severe treatment by the interrupted current effects a cure, but a small minority of patients fail to benefit by any of the usual methods, and often pass into a state of voicelessness which persists for years, and often for life.

Now the frightening of the voice back always seems to me on a par with frightening children into good behaviour ; it is uncertain in its effect, and relapse is common. It is only systematic treatment on definite lines which can be depended upon for any permanent effect, either in the case of a naughty child or the ill-behaving vocal cords ; and I venture to think that the treatment I now advocate is based upon sound principles, and can be successfully carried out by any medical man.

I have adopted it myself with success for the last four or five years, and so far as I can ascertain it is a method which has not hitherto been described for the cure of functional aphonia. Should I, however, be mistaken in my assumption, I must apologise for having inadvertently trespassed upon someone's preserves.

Before illustrating by specific cases, I would premise by saying that the treatment is based upon the fact that in almost every case there is some sort of a sound which can be produced by the approxi-

[1] Read at a Meeting of the Bristol Medico-Chirurgical Society at Weston-super-Mare, on June 12th, 1907.

mation of the vocal cords, and this note has, so to speak, to be caught and educated. As a rule, it is a very high-pitched note—C or D above—of the very feeblest quality, often not louder than a mouse's squeak, and inaudible at a few feet away. It requires great patience on the part of the doctor to catch and educate it.

In most cases the sound is more easily made with mouth almost or quite shut. As the voice strengthens, the patients can gradually open the mouth more and more, but any attempt to make them start with a big, round mouth—as a singing master would advise his pupils to do—is useless, and they themselves, as a rule, choose to keep the lips fairly close together. Although the first sound produced is usually very high-pitched, in one patient the only sound I could elicit was a low-pitched grunt—about F below middle C ; this is unusual, I believe. It is very desirable to ascertain on the piano the note the patient first pitches on, either as a squeak or as a grunt, and to record this note in a book, so as to start on the same note the next lesson.

Sometimes it is not easy to make out what note a grunt is, but you can get pretty close to it, and after a few trials the patient will attune her voice to the note you have decided on as being nearest to her first attempt. It is well to remember that it is easier to " hum " than it is to pronounce a definite vowel sound, as " o " or " e." At the first interview you strike middle C on the piano, telling the patient to try to sing or " hum " to it. You then strike D, E, F, G, A, B, C in quick succession. As a rule, with the lower notes no sound beyond a gasp or whisper is heard, but when upper B or C is reached the patient usually gives a momentary faint squeak, which, if prolonged, relapses at once into a whisper. At the first interview you must be contented with this momentary feeble squeak. On the following day start at once on the note the patient succeeded in producing the day before—say C above—at first, as yesterday, being satisfied with a succession of short, sharp, falsetto sounds, with intervals of rest between. After practising the interrupted sounds for a few minutes, it is well to hold the note on the piano down for a brief space, telling the patient to continue to squeak as long as it is held down. At first the sound is very tremulous, but by the end of the lesson, which lasts about ten minutes, it has usually become firmer, somewhat like a miniature siren. If it does not become firm, go back for a time to the interrupted squeaks. When the voice has thoroughly grasped the siren or humming sound, and can sustain it without breaking for a few seconds at a time, the vowel sound " o " should be attempted, and as soon as that has been accomplished a word, *e.g.* Do of the Do, Re, Mi should be learnt and sung on the same note. When the " Do " is first attempted it often results in failure, the voice breaking into a whisper again. Should that be the case, let the patient pronounce it as two words, De (short) O ; she will then whisper the first part, De, and sing the O, and after a few trials will arrive at Do as one word. By this time she will, as a rule, be able to take a lower note and a higher one, and to sing the words Do, Re, Mi up and down on these three notes till they are sung firmly and fairly loudly. If there is any difficulty let her repeat the Do up and down, and soon the words Re and Mi are learnt, as the voice strengthens and she gains confidence. If the patient has anything of a voice the scale can soon be managed, and after that, or before, if the patient is not a singer, words can be intoned, and in reply to your questions the answers must be intoned, so that the patient gradually learns to use a large number of different words. After that she can try a simple song, as " Three Blind Mice," repeating the same sentence several times running, and then pass on to intoning on a few notes any piece of reading you choose. When she can sing or intone easily anything you give her, it is time to start speaking, and after practising the singing voice for a few minutes tell her to read instead of singing, starting with a simple sentence and repeating it very slowly after you. If there is any hesitation, let her at once go back to the singing, and try again. It may require two or three attempts. After that she can practise reading aloud regularly at home, but should always keep the voice in working order by daily singing.

This is an outline of the treatment, which can be modified as occasion demands. It is very desirable while you are carrying out this treatment in a severe case to isolate the patient, particularly if she happens to be living in the bosom of a large and sympathetic family. If possible, let her stay with a nurse or any sensible woman who will carry out your instructions conscientiously. Strict orders should be given that she must not whisper at all, but after she has learnt to intone permission may be given to use her voice in conversation by intoning.

After the first two or three lessons, when she can sing Do, Re, Mi, it is a good plan for her to practise these first three notes twice or thrice a day for ten minutes at a time at the piano, her companion striking the notes and insisting on their being sounded firmly and steadily.

In the most severe case I had it took ten or eleven days before she could speak at all vigorously. In mild cases two or three visits may often suffice to restore the voice.

I will now illustrate by two cases, which I have taken as bad ones.

**Case 1.**—A French lady, a teacher, about 23 years of age, came to me on April 30th, 1902. She had lost her voice sixteen months ago, and had been treated at a throat hospital in town by electricity, &c. She had naturally a very weak voice, and had never sung. The only note she could produce was a high squeak on C above middle C, and B next below not so well. Both these notes were of the feeblest quality, barely audible a few feet away. On attempting notes above or below B or C the voice at once became a whisper. Three or four days later she succeeded in producing upper B, C, D fairly strongly, and also A and G very tremulously. I bade her practise these five notes—G, A, B, C, D—thrice daily, and enjoined on her the necessity of not trying to speak at all, but of asking for what she wanted in a sing-song on upper C. Three days after that she sang fairly clearly from G up to D, and at the same lesson spoke in a feeble voice. From that date she improved rapidly.

**Case 2.**—A young lady, age 19 years, came to see me June 18th 1904. She had lost her voice six months ago. She was very strong physically, and clever. Had had treatment, including electric battery, without benefit. Could only speak in a whisper. At the first interview the only sound I could elicit, after over a quarter of an hour's trial, was a very feeble, low-pitched grunt, about F below middle C, barely audible. No note in the nature of a high-pitched falsetto note could be produced at this visit. Three days later, on again making the grunt, the voice broke on to a momentary falsetto squeak, just audible, on E above upper C. On the following day she started at once on E above, and soon produced all the notes by humming from upper C down to middle C, but broke down at once into a whisper when told to sing Do. By pronouncing the Do as two words—De—O—she soon succeeded in singing it as one word. The next day she sang the octave, and in answer to various simple questions intoned the replies. On June 26th, eight days after her first visit, after singing the octave and various sentences clearly for a short time, she succeeded, with some difficulty, in speaking a simple sentence, repeating the words after me very slowly. From this time the voice got rapidly stronger, and I advised her to practise singing every day to keep the voice strong.

These two cases I have chosen as they had both been treated by experts in throats, and the battery had been applied in both cases without success.

